# Potential role of genomic imprinted genes and brain developmental related genes in autism

**DOI:** 10.1186/s12920-020-0693-2

**Published:** 2020-03-26

**Authors:** Jian Li, Xue Lin, Mingya Wang, Yunyun Hu, Kaiyu Xue, Shuanglin Gu, Li Lv, Saijun Huang, Wei Xie

**Affiliations:** 1 Key Laboratory of DGHD, MOE, Institute of Life Sciences, Southeast University, Nanjing, 210096 China; 2 Department of Bioinformatics, School of Biomedical Engineering and Informatics, Nanjing Medical University, Nanjing, 211166 China; 3 Foshan Women and Children Hospital, Foshan, 528000 China

**Keywords:** Autism, Imprinted gene, Brain development

## Abstract

**Background:**

Autism is a complex disease involving both environmental and genetic factors. Recent efforts have implicated the correlation of genomic imprinting and brain development in autism, however the pathogenesis of autism is not completely clear. Here, we used bioinformatic tools to provide a comprehensive analysis of the autism-related genes, genomic imprinted genes and the spatially and temporally differentially expressed genes of human brain, aiming to explore the relationship between autism, brain development and genomic imprinting.

**Methods:**

This study analyzed the distribution correlation between autism-related genes and imprinted genes on chromosomes using sliding windows and statistical methods. The normal brains’ gene expression microarray data were reanalyzed to construct a spatio-temporal coordinate system of gene expression during brain development. Finally, we intersected the autism-related genes, imprinted genes and brain spatio-temporally differentially expressed genes for further analysis to find the major biological processes that these genes involved.

**Results:**

We found a positive correlation between the autism-related genes’ and imprinted genes’ distribution on chromosomes. Through the analysis of the normal brain microarray data, we constructed a spatio-temporal coordinate system of gene expression during human brain development, and obtained 13 genes that are differentially expressed in the process of brain development, which are both autism-related genes and imprinted genes. Furthermore, enrichment analysis illustrated that these genes are mainly involved in the biological processes, such as gamma-aminobutyric acid signaling pathway, neuron recognition, learning or memory, and regulation of synaptic transmission. Bioinformatic analysis implied that imprinted genes regulate the development and behavior of the brain. And its own mutation or changes in the epigenetic modification state of the imprinted control region could lead to some diseases, indicating that imprinted genes and brain development play an important role in diagnosis and prognosis of autism.

**Conclusion:**

This study systematically correlates brain development and genomic imprinting with autism, which provides a new perspective for the study of genetic mechanisms of autism, and selected the potential candidate biomarkers for early diagnosis of autism in clinic.

## Introduction

Autism Spectrum Disorder (ASD) is a group of neurodevelopmental disorders characterized by deficits in social and verbal communication, and the presence of restricted interests, stereotyped and repetitive behaviors [1]. Epidemiological studies estimated the prevalence of ASD up to 1% [2]. The etiology of ASD has not been fully identified and lacks biological markers [3]. In clinic, it mainly relies on doctors’ observation of children’s behavioral characteristics and parents’ description for diagnosis, which is extremely difficult and subjective. Increasing evidences suggested that genetic factors, fetal intrauterine environment, and external environmental factors (i.e. nutrition and immunity) play important roles in autism [4]. For example, twin studies have shown that the comorbidity rate of autism in identical twins is higher than that of fraternal twins (60–80% VS 3–10%). Siblings of ASD patients have a 3 to 5% chance of regurgitation from the group. The prevalence of autism is 50 to 100 times [5]. A number of previous studies have made significant progress in search for autism susceptibility genes or pathogenic genes and mutations through genome-wide association studies (GWAS), whole-genome sequencing and exon sequencing [6, 7]. Among them, GWAS collects disease samples and normal controls to find genes or loci that are strongly associated with disease phenotypes by genome-wide genotyping. Wang et al. performed a GWAS and case-control association study on 780 families (3101 subjects) from Autism Genetic Resource Exchange (AGRE) and 8695 European individuals (1204 ASD vs 6491 control). Six single nucleotide polymorphisms (SNPs) located between cadherin 10 (*CDH10*) and calmodulin 9 (*CDH9*) on p14.1 region of chromosome 5 were found to be significantly associated with ASD and verified in two other independent samples [8]. Richard et al. performed a GWAS analysis of 1369 patients from the Autism Genome Project (AGP), including 1385 patients with ASD, and found that a SNP located within *MACROD2* was significantly associated with ASD. Unfortunately, the addition of 1301 family re-association analyses failed to replicate this result [9]. Due to the heterogeneity of the ASD phenotype and the relatively small sample size, the risk of variability in the autism GWAS study is less than that of other complex diseases, such as schizophrenia [10]. According to a recent investigation, the cause of ASD is mainly attributed to common mutations in the genetic structure, while rare new mutations lead to individual cases. Quantitative genetic estimates of the heritability of autism found that 52.4% were almost entirely caused by common variants, and only 2.6% were due to rare variants [11]. Studies have shown that about 7–20% of ASD individuals carry rare or new copy number variations (CNVs) [12]. In 2007, Sebat et al. performed high-resolution genomic microarray analysis of 264 families to detect the rate of de novo CNV in ASD patients and normal subjects. It was confirmed for the first time that de novo CNVs were significantly associated with autism (*P* = 0.0005). The author further validated de novo CNV by comparative genomic hybridization (CGH), cytogenetics, microsatellite genotyping and other techniques. A 1.1 Mb deletion was detected on p13 region of chromosome 20 in children with Asperger syndrome, which involved 27 genes, including the oxytocin gene OXT [13]. Copy number variants in autism patients’ genome imply the role of the involved genes in the pathogenesis of autism. Mutations also impair the function of the related genes to lead to autism. Until 2019 March, SFARI database collected 1045 autism susceptibility genes. However, single gene mutations merely account for 1 to 2% of autism cases, revealing the high degree of genetic heterogeneity in autism [14, 15]. ASD is usually associated with other diseases, such as mental retardation, motors defect, sleep disorders, gastrointestinal disorders and epilepsy [16]. For the current study, the etiology of autism has not been completely determined and lacks of biological markers [14]. In particular, the imperfection of early diagnosis of autism hinders the improvement of clinical treatment and prognosis for patients [17]. So far, in clinic, the diagnosis of autism mainly relies on doctors to observe the behavioral characteristics of children and the description of parents about the children. Such diagnosis is extremely difficult and subjective, and even most early diagnosis only can be done until 24 months at the earliest [18].

Genomic imprinting is an epigenetic phenomenon, in which genes present in a single allele expression depending on the source of the parents [19]. Many imprinted genes have different expression patterns and functions in brain and other tissues [20]. Previous studies have shown that most of the imprinted genes can express in brain and placenta, which are proved to regulate the process of placental development and fetal growth [21]. An increasing number of researches have suggested that imprinted genes also play important roles in the postnatal stage, such as neonatal feeding, regulating metabolism, sleep and the behavior of mother care [21]. Deregulation of imprinted genes expression can cause some clinical syndromes (i.e. intrauterine growth restriction (IUGR), obesity, diabetes, mental disorder) [22]. As a cognitive and developmental disorder, the clinical phenotype of autism is similar to the imprinting syndrome angel syndrome (AS) and prader-willi syndrome (PWS) [21], suggesting that there may be a certain relation between genomic imprinting and ASD.

The progress of neuroimaging techniques enables positron emission tomography (PET), magnetic resonance imagining (MRI), functional magnetic resonance imagining (fMRI) and other technologies to be widely applied in brain-related studies. Brain imaging studies have found that patients with autism have heterogeneous disorder or deformity [23, 24]. The study found that 92–100% of ASD individuals have cortical hypoplasia, of which 31% cases occurred in subcortex, ventricle, hippocampus and cerebellar ectopic and 61% of subjects showed cerebellar hypoplasia. The unanimous conclusion from studies of brain development in ASD is that the head size is normal at birth, but significantly enlarged at age 2–3 [24, 25]. Brain imaging studies of at-risk infants with ASD also show that excessive expansion of the cerebral cortex surface area during 6–12 months leads to overgrowth in brain volume in the second year. Consequently, autism cannot be diagnosed until the 24th months at the earliest [26]. The overgrowth of the brain volume is related to the occurrence and severity of autism social defects. It indicates that autism had a close relationship with neuron function during brain development.

In this study, we firstly collected all the reported autism-related genes, and analyzed the expression of autism-related genes in the scenario of normal brain developmental process and genomic imprinting. Then, we explored the correlation among them, aiming to explain the pathogenesis of autism from the perspective of brain development and genomic imprinting. Eventually, we hope to provide potential biomarkers for genetically early diagnosis of autism in clinic.

## Methods

### Collect and collate data

#### Collect and collate autism-related genes

Autism-related genes were collected from NCBI (National Center for Biotechnology Information) (https://www.ncbi.nlm.nih.gov/gene), SFARI Gene (the database of SFARI (Simons Foundation Autism Research Initiative)) (https://gene.sfari.org/database/human-gene/) and HGMD (Human Gene Mutation Database) (http://www.hgmd.cf.ac.uk/ac/index.php). We used retrieval condition: (autism) AND “*Homo sapiens*” [porgn:__txid9606] in NCBI database. The SFARI Gene database is an online database of genes implicated in autism susceptibility. We used the Human Gene module of the SFARI Gene to download all the known human genes associated with autism. For the HGMD database, we purchased the HGMD professional which is a commercial version. We searched for the autism genes by keywords “autism” and “autism spectrum disorder”. Then we downloaded the RefSeq annotation file from UCSC database (https://genome.ucsc.edu/cgi-bin/hgTables). We extracted the information with Perl language, including gene terminology, gene ID, genome mapping (the chromosome number and the starting position), etc. Through GeneCards (https://genecards.weizmann.ac.il/v3/) and Ensembl database (http://grch37.ensembl.org/index.html), we improved the annotation information about autism-related genes involving mutation genes, mutation type and related supporting documents (Fig. S1). Finally, we obtained 1905 autism genes (Table S1).

#### Collect and collate imprinted genes

Human imprinted genes were collected from geneimprint database (http://www.geneimprint.com/site/genes-by-species) with the retrieval condition: genes→genes by species→Human. Finally, we obtained 244 imprinted genes after verification by the same method as above.

#### Collect and collate microarray data of normal human brain gene expression

By searching the published document in the PubMed (https://www.ncbi.nlm.nih.gov/pubmed/) and Gene Expression Omnibud (GEO) database (https://www.ncbi.nlm.nih.gov/geo/) under NCBI, we finally chose brain expression profile (registry number: GSE25219, https://www.ncbi.nlm.nih.gov/geo/query/acc.cgi) as a set of original data of this study.

The experimental material for this dataset is based on 16 brain regions of 57 human brain specimens (Table S2), which includes cerebellar cortex, thalamus, amygdala, striatum, hippocampus and 11 new cerebral cortex regions. Gene expression data of 1340 brain tissue samples were analyzed by using Affymetrix’s HuEx-1_0-st chip. With a range of 5.7 weeks to 82 years of donors’ age, this study established a continuous brain developmental process containing 15 stages from embryonic development to late adulthood based on the time points of major neurodevelopmental events in the human brain (Table S3).

### Functional enrichment analysis of autism-related genes

In this study, enrichment analysis of functions and signaling pathways of the collected autism-related genes was conducted by the Over Representation Analysis (ORA) method using all the genes in the human reference genome as annotation sets. ORA method intersects the list of genes of interest (usually differentially expressed genes) with the functional set to be tested and counts the common genes and differential genes. Then the statistical analysis method is used to evaluate whether the observed value is significantly higher than the random value. That is to say, whether the functional set to be tested is significantly enriched in the gene list. The statistical test method is a hypergeometric distribution with a significant cutoff *P*-value set to 0.05.

We used R language for enrichment analysis of autism genes. Origin is a scientific mapping and data analysis software developed by OriginLab. In this study, the enrichment analysis result figures were drawn by origin8.5.

Enrichment analysis can reduce the complexity of the analysis and uncover key biological pathways in biological processes. This study adopted Gene Ontology (GO) to conduct enrichment analysis of functions and Kyoto Encyclopedia of Genes and Genomes (KEGG) pathway to conduct enrichment analysis of pathways.

### Correlation analysis between autism-related genes and imprinted genes

We downloaded cytoBand.txt.gz from UCSC database (http://hgdownload.soe.ucsc.edu/goldenPath/hg38/database/). The cytoband.txt file contains cytogenetic g-band data, which can distinguish different regions of chromosomes. In this study, we used the GD module (the website dynamic graphics generation tool) of Perl language referenced the cytoband.txt file to locate the autism-related genes and imprinted genes respectively on the chromosomes for visualization. In order to study the correlation between autism-related genes and imprinted genes distribution on all chromosomes, we used the sliding window method to count the reference genome-annotated genes, autism-related genes and imprinted genes in each window (Fig. S2). The correlation analysis of autism-related genes and imprinted genes was implemented by R language. The result figure was draw by origin8.5.

The number of the autism-related genes and imprinted gene distribution on the chromosome was subjected by Fisher’s exact test. The sliding window was implemented by Perl programming.

AutoCAD (Autodesk Computer Aided Design) is a automatic computer-aided design software first developed by Autodesk in 1982. It is used for two-dimensional drawing, detailed drawing, design documents and basic three-dimensional design. Now it has become a widely popular drawing tool in the world. In this study, we used it to draw the model of sliding window.

### Expression profiles analysis of normal human brain genes

#### Microarray data preprocessing

Original data of normal human brain microarray was converted hybridization signal into the expression value through preprocessing. Affy microarray data preprocessing usually involves three steps: background adjustment, normalization and summarization.

The RMA method estimates the mean value of the non-specific hybridization background of the chip by the cyclotron model, and the PM probe value subtracts this mean value for correction, while the MM probe is not processed. This study used RMA method to run background adjustment. Since the microarray data belongs to Affymetrix Human Exon 1.0 ST Array, which was conducted by Oligo package of R software (version number: 3.4.3).

Then, the quantile method assumes that the empirical distribution function of each chip probe signal should be exactly the same, and the QQ graph of any two chip data should get a line with slope 1 and intercept 0. After normalization by quantile method, the dispersion degree of each sample was at the same level and was comparable.

Lastly, we used appropriate statistical methods to convert hybridization signals from the probe set into gene expression values. The medianpolish method was used for summarization.

#### Differential expression screening of microarray data

We compared genes expression of normal human brains from two angles (i.e. the same brain region in adjacent time and the same time in any two brain regions). This study used the non-parametric empirical Bayes method to select differentially expressed genes and this method can be implemented by the limma package of R. Screening of differentially expressed genes: adj.*P-*value< 0.05 and |Fold Change (FC)| ≥ 2.

### Functional analysis of normal human brain differentially expressed genes, imprinted genes and autism-related genes

This study intersected autism-related genes and imprinted genes with brain differentially expressed genes, which were collected previously.

#### Enrichment analysis of genes

We analyzed the common genes of the above three by the ClusterProfiler of R to conducted GO and KEGG analysis with cutoff value of *P*-value is 0.05.

#### Expression trajectory analysis of common genes

In order to illustrate the dynamic change of spatial and temporal expression of genes, we plotted gene expression trajectory with a single expression value for a specific brain region at a specific time, which was the average of gene expression in a brain-related sample at a certain time. This study plotted gene expression trajectory by origin8.5 and Perl script.

#### Prediction of intergenic interaction

We predicted intergenic interaction of normal human brain differentially expressed genes, imprinted genes and autism-related genes by GeneMANIA tool of Cytoscape (version number: 3.6.0). We selected *Homo sapiens* (H.sapiens) as species, and built up the interaction network for their common genes using Cytoscape.

## Results

### Functional enrichment analysis of autism-related genes

**Fig. 1:**
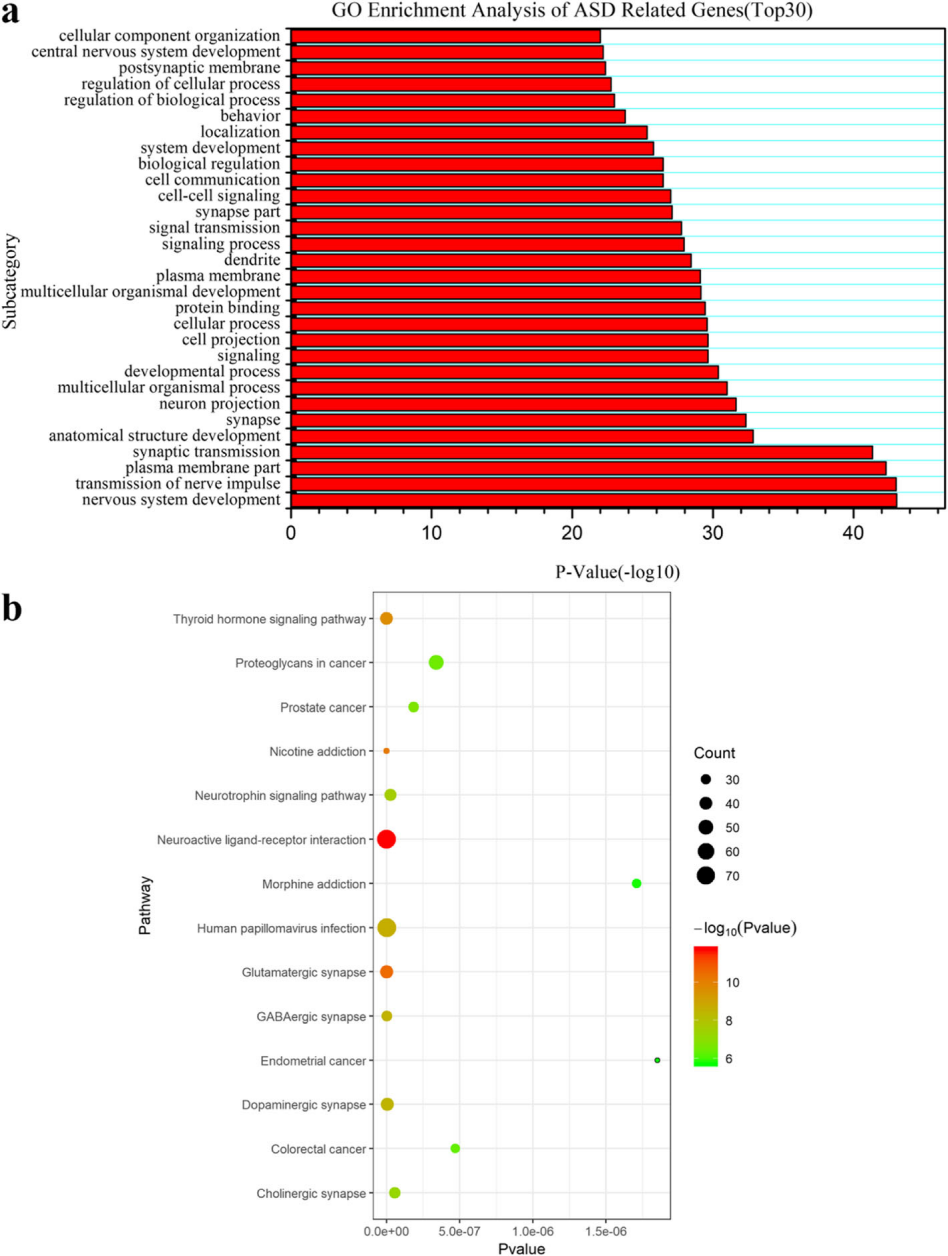
GO and KEGG analysis of autism-related genes. **a.** GO analysis of autism-related genes. It listed the top 30 biological processes in the results. **b.** KEGG pathway analysis of autism-related genes. Circle: the proportion of the number of genes in the pathway to the total number of gene sets. The larger the circle, the more products are enriched. Color: the size of enriched -log_10_ (*P*-value). The greener the color, the smaller the -log_10_ (*P*-value)

This study systematically collected and collated 1905 autism-related genes, which have completed annotation information (Table S1). Enrichment analysis of GO (Fig. 1a and Table S4) showed that autism-related genes participated in multiple biological processes, mainly including synaptic transmission and regulation, nervous system development, gated channel activity, signal transduction, learning and memory and the brain development. KEGG (Fig. 1b) analysis revealed that 111 pathways, such as Calcium, mTOR, Wnt, Notch signal pathway, neuroactive ligand-receptor interaction, neurotrophin signaling pathway and cancer, were involved. Figure 1b shows the top 14 most significant pathways of the 111 pathways, the rest enriched pathways were presented in supplementary file (Table S5). Notably, the distribution of autism-related genes on chromosomes was not random. The number of autism-related genes on chromosome 2, 3, 7 and X was significantly abundant and enriched than that on chromosome 14, 19 and Y (Table 1). From the view of the related diseases, we found these autism-related genes were not only enriched in autism, schizophrenia or other mental illness, but they were also associated with attention deficit, hyperactivity, bulimia, kidney disease, homocysteinemia and other diseases (Table 2).

**Table 1:** Chromosome enrichment analysis of autism-related genes

Category	Subcategory	Expected	Observed	*P*-value	Enrichment
Chromosomal Location	Y	12.6335	2	0.0061757	down
Chromosomal Location	14	45.1195	25	0.00693292	down
Chromosomal Location	X	54.0431	75	0.0229711	up
Chromosomal Location	3	55.121	75	0.0271095	up
Chromosomal Location	2	70.4616	92	0.0271095	up
Chromosomal Location	19	59.3071	42	0.0361538	down
Chromosomal Arm Location	2q	40.0111	67	0.0101119	up
Chromosomal Arm Location	14q	45.4507	25	0.0101119	down
Chromosomal Arm Location	3p	26.1187	42	0.0204816	up
Chromosomal Arm Location	Xp	20.5659	35	0.0204816	up
Chromosomal Arm Location	7q	38.3407	56	0.0277397	up
Chromosomal Arm Location	19q	36.1372	22	0.046029	down

**Table 2:** The results of enrichment of autism-related diseases

Category	Subcategory	Expected	Observed	*P*-value
NIA human disease gene sets	Autistic Disorder	3.84196	27	2.25E-20
NIA human disease gene sets	Mental Disorders	33.3853	87	2.25E-20
NIA human disease gene sets	Schizophrenia	19.8722	52	7.73E-11
NIA human disease gene sets	Mood Disorders	2.38467	12	0.0000174
NIA human disease gene sets	Bipolar Disorder	7.81641	23	0.0000261
NIA human disease gene sets	Substance-Related Disorders	2.25219	11	0.0000615
NIA human disease gene sets	Attention Deficit Disorder with Hyperactivity	4.10693	15	0.0000911
NIA human disease gene sets	Obsessive-Compulsive Disorder	1.32482	8	0.00013362
NIA human disease gene sets	Nervous System Diseases	55.3773	82	0.00032166
NIA human disease gene sets	Alcoholism	6.22663	18	0.00036562
NIA human disease gene sets	Psychotic Disorders	2.38467	10	0.00073627
NIA human disease gene sets	Disorders of Environmental Origin	19.8722	37	0.00133675
NIA human disease gene sets	Bulimia	1.32482	7	0.00152108
NIA human disease gene sets	Neurotic Disorders	1.4573	7	0.00344979
NIA human disease gene sets	Antisocial Personality Disorder	0.794889	5	0.00471419
NIA human disease gene sets	Heroin Dependence	0.794889	5	0.00471419
NIA human disease gene sets	Anxiety Disorders	1.58978	7	0.00600006
NIA human disease gene sets	Tourette Syndrome	1.19233	6	0.00600006
NIA human disease gene sets	Tobacco Use Disorder	3.04707	10	0.00606997
NIA human disease gene sets	Alzheimer Disease	14.8379	28	0.00613592
NIA human disease gene sets	Depressive Disorder	4.37189	12	0.0100685
NIA human disease gene sets	Parkinson Disease	6.22663	15	0.0100914
NIA human disease gene sets	Eating Disorders	0.927371	5	0.0102424
NIA human disease gene sets	Kidney Diseases	2.78211	9	0.0110736
NIA human disease gene sets	Conduct Disorder	0.662408	4	0.0194026
NIA human disease gene sets	Autistic Disorder	3.84196	27	2.25E-20

Subcategory column: enriched disease species; Expected: the number of theoretically ASD-related genes; Observed: the number of genes actually enriched; *P*-value: the *P* value obtained by the hypergeometric distribution method (all *P*-value < 0.02 above)

### Correlation analysis between autism-related genes and imprinted genes

**Fig. 2:**
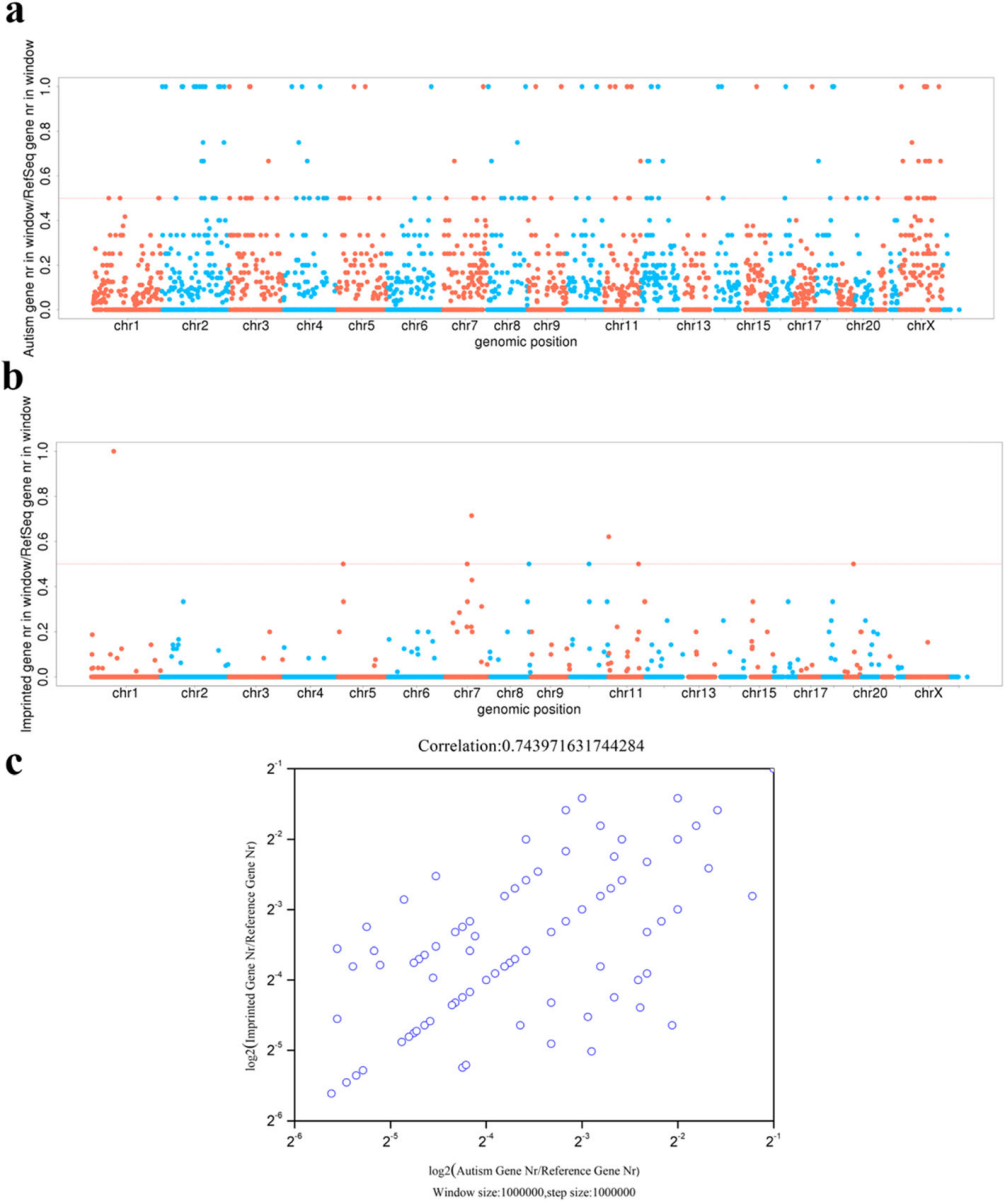
Correlation analysis between autism-related genes and imprinted genes. The sliding window size is 1000 kb and the moving step size is 1000 kb. **a.** The distribution of autism-related genes on the genome. It represents the ratio of the number of autism-related genes in each window to the number of reference genes and sets the dividing line at 0.5 points. **b.** The distribution of imprinted genes on the genome. It represents the ratio of the number of imprinted genes in each window to the number of reference genes and sets the dividing line at 0.5 point. **c.** Logarithmic plot between autism-related genes and imprinted genes. After counting the number of reference genes, autism-related genes and imprinted genes in each window, the ratio of the two to reference genes is taken

In order to study the correlation between autism and genomic imprinting, we positioned the collected autism-related genes and imprinted genes into chromosomes respectively. Visualization of imprinted genes and autism-related genes has shown that imprinted genes were highly clustered on chromosomes (Fig. S3b), and autism-related genes also widely enriched on chromosomes (Fig. S3a). We observed a positive correlation between the two group genes on all chromosomes with the correlation coefficient was 0.74 (Fig. 2c), depending on countable analysis of related genes by sliding window method. In addition, Fig. 2a illustrated that the ratio of autism-related genes to reference genes was 1 on chromosome 2 and X, indicating that all genes in these two chromosomes were related to autism-related genes. However, there was no significant correlation in the whole genome between the imprinted genes and human genome reference genes using the same method (Fig. 2b).

### Expression analysis of normal human brain genes

**Fig. 3:**
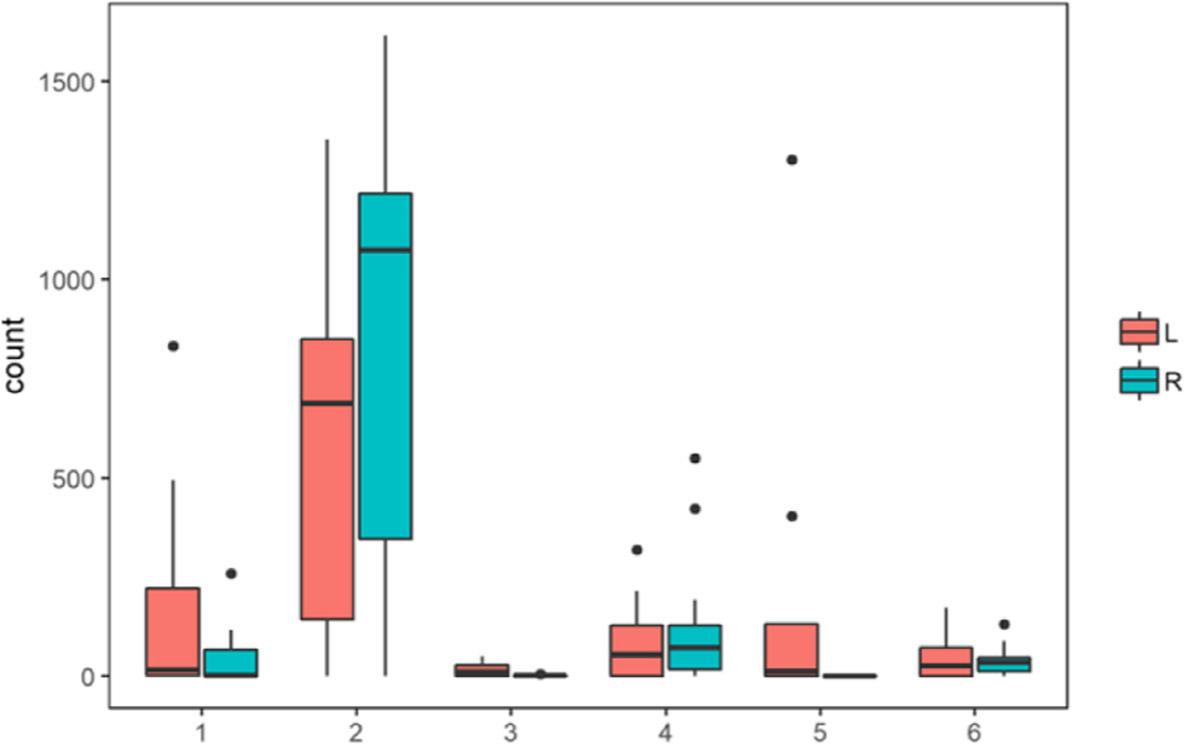
Expression profiles analysis of normal human brain genes. The distribution count of brain differentially expressed genes in the adjacent time. Comparison of adjacent periods in the same brain region, the number of all brain regions in the adjacent period is counted as a box plot, with red for the left brain and blue for the right brain. The x axis represents different stages of development. 1: stg1-stg2; 2: stg2-stg3; 3: stg5-stg6; 4: stg6-stg7; 5: stg10-stg11; 6: stg14-stg15. The specific development stages are shown in Fig. S2

We constructed a spatio-temporal coordinate system of gene expression by re-analysis of the brain gene expression microarray data. Figure 3 told the distribution of differentially expressed genes at adjacent time points in the same brain region were mainly expressed in the following four stages: early embryo development to early fetal development, mid-late fetal development to prenatal stage, early childhood to childhood and middle age to old age. While the comparison of the same time points in any two brain regions suggested that the distribution count of differently expressed genes followed a rule: the trend of the number of differentially expressed genes varied from regions. They were less distributed among 11 cerebral cortical regions, however there were significant differences between the hippocampus, amygdala, cerebellum, thalamus, and striatum and other 15 brain regions (Table 3).

**Table 3:** Distribution of differentially expressed genes in any two brain regions during the same period

NCX																		
NCX			OFC	DFC	VFC	MFC	M1C	S1C	IPC	A1C	STC	ITC	V1C	HIP	AMY	CBC	MD	STR
	FC	OFC			1			1		36	22	44	13	449	587	614	867	545
		DFC					16	8		51	47	86	9	690	829	826	991	603
		VFC				10						1	39	614	677	808	880	525
		MFC	14	11	6		6	9	2	46	36	91	1	574	710	806	943	485
		M1C	13	27	2	12					1	6	2	743	841	945	998	618
	PC	S1C	40	57	2	34						1		729	882	883	974	626
		IPC	78	91	40	71	2	2						252	235	196	481	219
	TC	A1C	87	97	55	99	29	1					3	603	634	770	923	509
		STC	91	105	64	102	30	5	1					422	429	776	985	369
		ITC	56	96	60	80	23	13	16				4	345	340	420	751	349
		V1C	137	153		178	95	117	92	113	107	103		156	229	516	960	377
		HIP	569	617	566	492	547	657	650	666	639	420	521		136	658	1077	568
		AMY	214	395	322	254	150	226	402	336	223		117	121		320	789	63
		CBC	889	977	936	867	946	1058	1013	1029	1029	843	808	565	380		580	346
		MD	826	862	770	732	711	793	845	849	859	677	929	443	574	745		588
		STR	691	732	708	623	655	731	778	744	708	567	562	475	207	599	641	

The upper right and lower left number represent the numbers of differentially expressed genes obtained in the brain regions of the third period (early fetal period) and the sixth period (fetal middle period), respectively. Different rows and columns represent different brain regions, and NCX represents 11 cerebral cortical regions

### Functional analysis of normal human brain differentially expressed genes, imprinted genes and autism-related genes

We intersected autism-related genes and imprinted genes with brain differentially expressed genes and obtained 13 common genes: *GABRA5, GABRG3, NTM, SNRPN, OTX1, FOXG1, TSHZ3, CDH18, GABRB3, GATM, HTR2A, DHCR7* and *NLRP2* (Table S6).

#### Enrichment analysis of the common genes

**Fig. 4:**
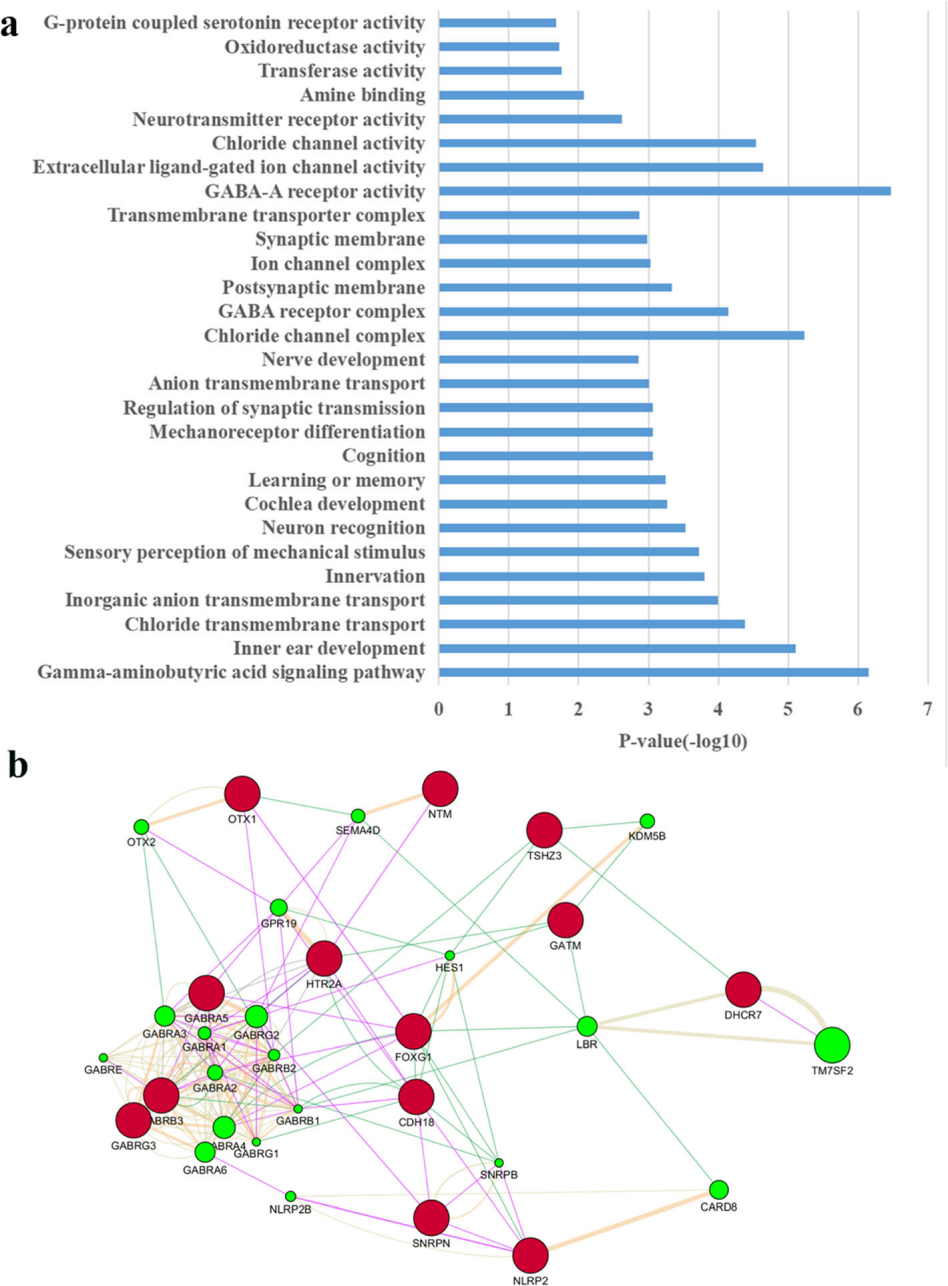
Analysis of brain differentially expressed genes, imprinted genes and autism-related genes. **a.** GO analysis of common genes of brain differentially expressed genes, imprinted genes and autism-related genes (*P*-value = 0.05). **b.** The reaction network of common genes of brain differentially expressed genes, imprinted genes and autism-related genes. Nodes (circles) represent the different genes, where the nodes labeled with red represent the input genes, and the nodes labeled with green indicate the genes that interact with the input genes. Connection (edge) indicates that there is an interaction relationship between them, and different colored edges represent different interaction types

GO analysis of the common genes showed (Fig. 4a) that they mainly enriched in some biological process, such as gamma-aminobutyric acid signaling pathway, inner ear development, sensory perception, learning or memory, regulation of synaptic transmission. Cell locations of these genes mainly are involved in GABA receptor complex, the postsynaptic membrane, ion channel complexes and transmembrane transporter complexes. While the most notable molecular functions were GABA-A receptor activity and extracellular ligand-gated ion channel activity.

KEGG pathway analysis found that these genes were mainly related to nicotine addiction signal pathway, GABA synaptic pathway, neuroactive ligand-receptor interaction signal pathway, serotonin synaptic pathway, steroid biosynthesis signal pathway (Table 4).

**Table 4:** KEGG analysis of common genes of brain differentially expressed genes, imprinted genes and autism-related genes

ID	Description	GeneRatio	BgRatio	*P*-value	*P*-adjust	qvalue	Gene symbol
Has05033	Nicotine addiction	3/8	40/7376	8.12E-0.6	0.000122	5.98E-0.5	*GABRA5/GABRG3/GABRB3*
Has04727	GABAergic synapse	3/8	88/7376	8.80E-0.5	0.00046	0.000226	*GABRA5/GABRG3/GABRB3*
Has05032	Morphine addiction	3/8	91/7376	9.73E-0.5	0.00046	0.000226	*GABRA5/GABRG3/GABRB3*
Has04080	Neuroactive ligand-receptor interactiom	4/8	278/7376	0.000123	0.00046	0.000226	*GABRA5/GABRG3/GABRB3/HTR2A*
Has04723	Retrograde endocannabinoid signaling	3/8	148/7376	0.000412	0.001235	0.000607	*GABRA5/GABRG3/GABRB3*
Has04726	Serotonergic synapse	2/8	113/7376	0.006133	0.015332	0.007532	*GABRB3/HTR2A*
Has00100	Steroid biosynthesis	1/8	19/7376	0.020432	0.043783	0.021508	*DHCR7*

Id: the number of the path in the kegg database; Description: enriched pathway; GeneRatio: the ratio of the number of genes enriched in the pathway to the total number of genes recognized by the database; BgRatio: the ratio of the number of genes associated with the id to all bg genes; *P*-value: statistically significant level; Gene symbol: the gene symbol of the gene involved in this pathway

#### Expression trajectory analysis of the common genes

We found 13 common genes that had different expression patterns spatially and temporally. As Fig. S4 shown, *CDH18* expression was significantly distinct in different brain regions, especially in cerebellum and striatum. *FOXG* expression pattern was polarised, which had low expression levels in the cerebellum and thalamus region, but higher expression levels in other regions. Likewise, *GABRA5* had similar expression pattern, which had lower expression level in cerebellum, compared with other regions. Furthermore, we also found the expression levels of 11 neocortical regions were similar in different periods and their trends in each period were fairly consistent by observing the 13 genes Expression trajectory (Fig. S4).

#### Prediction of intergenic interaction of the common genes

Multiple genes normally coordinate with each other. The phenotype of organisms can be impacted by the interaction among different genes. Molecular biological network can show the interaction of different molecules and regulatory relationship among genes expression. Figure 4b shows that *FOXG1* had direct co-expression relationship (Purple) with *OTX1* and *GABRA5*. *CDH18* had same expression pattern with *SNRPN*, *NLRP2* and *HTR2A*. While *HTR2A* had direct genetic interaction (Green) with *GABRB3*, *CDH18* and *GATM*, so do *FOXG1*, *CDH18* and *NLRP2*. Merely, there was a co-localization between *GABRA5* with *GABRB3*, *GABRA1*, *GABRA3*, *GABRA4* and *HTR2A* (Grey). The result indicated that these genes interacted with each other. We could associate imprinted genes with brain development and autism.

## Discussion

This study systematically collected 1905 autism-related genes. Enrichment analysis of GO and KEGG have shown that autism-related genes involved in more than 1700 molecular functions, cellular localization, biological processes and 111 signal pathways. Most of them were highly correlated with synaptic transmission and regulation, anatomical structural development, and the nervous system development. Autism-related genes were significantly associated with attention deficit, hyperactivity, bulimia, kidney disease, homocysteinemia and other diseases. Surprisingly, they were enriched in mental illness, such as autism, schizophrenia and mood disorders, which suggests that parts of autism-related genes were related to metabolic diseases, but not causative genes. Thus, we speculated that these genes could not cause autism-related genes core symptoms. Additionally, this result was consistent with a clinical finding that most patients suffering ASD were associated with a variety of other disorders, including developmental disorders, nutritional problems, eating behavioral problems, gastrointestinal disorders, sleep disorders, and epilepsy [16].

A large amount of imprinted genes had shown significant function in maintaining placental development, regulating fetal growth, metabolism, neurological function and social behavior. There have been studied that imprinted genes abnormally expressed would be associated with parts of cognitive diseases, one of which was autism. A typical example is the partial deletion of q11–13 on chromosome 15 that leads to AS and PWS. The phenotype of syndrome varied from paternal mutations and maternal mutations [21]. In order to analyze the correlation between autism and genomic imprinting, we investigated the locations of the collected autism-related genes and imprinted genes on chromosomes and found that most of the imprinted genes were clustered. Based on the phenomenon of significant enrichment of autism-related genes on chromosome 2, 3, 7 and X, we further found a positive correlation between the two group genes on chromosomes using sliding window. Meanwhile, genes in certain regions of chromosome 2, 11 and Y were all autism-related genes, which was consistent with previous analyses of the enrichment of autism-related genes.

Multiple studies have revealed that autism belonged to brain development disorder. The neuropathological studies consistently found that the brain of autism patients had different types of malformations, including cortical dysplasia, hippocampus and cerebellar ectopic, cerebellar hypoplasia [27]. In addition, brain imaging study also suggested that excessive growth of the brain capacity was related to the occurrence of autism and the severity of social defects [28–32]. All of the above results indicated that there was a close relationship between autism and brain development. Therefore, what is the specific connection between them? Do gene expression abnormality in brain regions at critical time of brain development cause autism?

We re-analyzed the microarray data of normal human brain. Through comparison of the 15 brain developmental stages, we revealed that the four stages with the largest number of differentially expressed genes compared with the previous stage during brain development were: early embryonic development to early fetal development, mid-late fetal development to prenatal stage, early childhood to childhood and middle age to old age. Furthermore, the distribution count of differentially expressed genes was discrete in early fetal development. It indicated that there was significant difference in different brain regions at this stage. Through the comparison of any two brain regions, we found that the gene expressions of the 11 cerebral cortical regions are quite similar, reflecting the similarity of their cell types and functions. Whereas, there were significant differences in gene expression among the following regions: cerebellum, hippocampus and other brain regions. This result confirmed that different brain structures follow different developmental and maturation trajectories in childhood and adulthood development [33, 34]. In spite of human brain matures until 25 years old [35], the biggest fluctuation occurred to infancy and early childhood. Interestingly, most neurodevelopmental diseases can be diagnosed in childhood and adulthood development. Moreover, clinical studies have shown that ASD can be diagnosed as early as 18–24 months. This result indicated that autism was related to brain development, which can provide a new clue for early diagnosis of autism patients.

To explore the impact of brain development and genomic imprinting on autism-related genes, we intersected autism-related genes with the constructed brain differential expression coordinate system and imprinted genes. Finally, we obtained 13 common genes: *GABRA5, GABRG3, NTM, SNRPN, OTX1, FOXG1, TSHZ3, CDH18, GABRB3, GATM, HTR2A, DHCR7* and *NLRP2*. Then, we performed an enrichment analysis of the common genes, aiming to uncover the key biological pathways among autism, brain development and genomic imprinting. From the enrichment analysis, we discovered that the common genes were mainly involved in some biological processes, such as GABA receptor complex, the postsynaptic membrane, gamma-aminobutyric acid signaling pathway, the inner ear development, sensory perception, learning or memory and regulation of synaptic transmission. This finding confirmed the conclusion that weighted gene co-expression network analysis (WGCNA) showed that autism risk genes were co-expressed in human embryonic mid-cortical neurons and pooled in the synaptic growth pathway [36]. In order to explore the expression of the common genes in the normal development of brain, we plotted these genes expression trajectory map. Figure S4 showed that each gene had different change patterns in development. Compared with other brain regions, gene expression pattern of epencephalon was very unique. The expression levels of the 11 neocortical regions of the common genes were similar in different periods, whose trends in each period were fairly consistent. This is consistent with the number of previously differentially expressed genes in brain regions. A typical example is the expression of *FOXG1* and *GABRA5*, which expressed low level in the cerebllum compared with other brain regions. Transcriptomic studies of 20 different regions in central nervous system (CNS) and 45 other organizations found that the expression patterns of the CNS regions are similar to each other but significantly different from other non-CNS organizations [37]. This result was consistent with our analytic results.

Imprinted genes were usually clustered on the chromosome. This feature allowed them to share regulatory elements, namely imprinted control regions (ICRs) [38]. Imprinted genes regulate the development and behavior of the human brain. A change in the epigenetic modification state of the imprinted control region or the self-mutation would cause disease. Previous studies have reported that mothers with *NLRP5* mutations had offspring with characteristic clinical features and disorders, such as fertility impairment, infertility, idiopathic developmental delay and autism. Mutations of other *NLRP* family genes, such as *NLRP7* and *NLRP2*, in parent’s genome result in familial hydatidiform mole and multisite imprinting disorder, respectively [39]. Besides, mutations in the imprinted gene *NLRP2* were also found in the germ lines of patients with beckwith-wideman syndrome (BWS), which also has been revealed in our analysis [40]. Interestingly, we found that *GABRA5, GABRG3, GABRB3* and *SNRPN* of common genes all locate in 15q11.2-q12 imprinted region. This gene cluster also includes *UBE3A, ATP10A, NDN, ZNF127* and other imprinted genes, which are related to PWS and AS [21]. The ICR is composed of the promoter of *SNURF-SNRPN* and the first exon. The paternal allele is methylated and the maternal allele is not, which controls the expression of *SNORD, IPW* and *UBE3A-ATS* [41]. Three-dimensional fluorescence in situ hybridization analysis showed that *GABRB3* was closer to *SNRPN* than *UBE3A* in three-dimensional structure, which suggests the influence of epigenetic regulation on gene expression [42]. Moreover, *SNRPN* as a part constituted 15q11.2-q12 imprinting control region, which could regulate the expression of the *GABRA5, GABRG3, GABRB3* and other genes. *GABR (GABRA5, GABRG3* and *GABRB3*) play important roles in the early stages of brain development. Previous studies have shown that it was closely related to autism and epilepsy [43].

## Conclusion

This study analyzed the correlation between the expression of autism-related genes and imprinted genes in the course of brain development. We propose the possibility for an association between autism and disorder of genomic imprinting in brain development. Thus, our research provided a clue for exploring the correlation of brain development, genomic imprinting and autism and offered potential biomarkers in early diagnosis of autism.

## Supplementary information

**Additional file 1:** **Figure S1.** The screening process of autism-related genes. The 512 autism-related genes were collected from NCBI using retrieval condition: “autism” AND “*Homo sapiens*” [porgn:__txid9606]. We collected 990 and 1348 autism-related genes from SFARI Gene database and HGMD database, respectively. We annotated these genes with the information of gene terminology, gene ID, genome mapping, mutation genes, mutation type and related supporting documents, etc., from GeneCards and Ensembl databases.

**Additional file 2:** **Figure S2.** Sliding window schematic diagram. The length of each chromosome was known and the position information of the genes on the chromosomes has been annotated. Setting a w size window, which slided from the starting position to the end on the chromosomes, step by step, until whole chromosome was traversed.

**Additional file 3:** **Figure S3.** The distribution of imprinted genes and autism-related genes on chromosome 1~Y. **a.** Distribution of imprinted genes on chromosomes 1~ − 22 and chromosomes X - Y. **b.** Distribution of autism-related genes on chromosome 1~ − 22 and chromosomes X - Y.

**Additional file 4:** **Figure S4.** Expression trajectory plot of 13 common genes. **a-c.** The first line from left to right is *CDH18, DHCR7* and *FOXG1*, respectively. **d-f.** The second line from left to right is *GABRA5, GABRB3* and *GABRG3*, respectively. **g-i.** The third is *GATM, HTR2A* and *NLRP2*, respectively. **j-l**. The fourth line is *NTM*, *OTX1* and *SNRPN*, respectively. **m.** The last line is *TSHZ3*. X axis: the 15 developmental periods; Y axis: the expression levels of different genes; The different colored lines indicate the expression of the same gene in different brain regions.

**Additional file 5:** **Table S1.** The autism gene list.

**Additional file 6:** **Table S2.** The name of brain and cortical areas.

**Additional file 7:** **Table S3.** The definition of human development stage.

**Additional file 8:** **Table S4.** Go analysis results of autism genes.

**Additional file 9:** **Table S5.** KEGG analysis results of autism genes.

**Additional file 10:** **Table S6.** The common genes of normal human brain differentially expressed genes, imprinted genes and autism-related genes.

## Data Availability

All autism-related genes involved in this manuscript are available in NCBI (https://www.ncbi.nlm.nih.gov/gene/?term=(autism)+AND+%22Homo+sapiens%22%5Bporgn%3A__txid9606%5D), and SFARI Gene (https://gene.sfari.org//wp-content/themes/sfari-gene/utilities/download-csv.php?api-endpoint=genes). HGMD professional is a commercial database and the homepage is http://www.hgmd.cf.ac.uk/ac/index.php. And then, we downloaded the RefSeq annotation file from the UCSC database. Subsequently, a perl script was used for gene information extraction, including gene terms, gene ID, genome map, etc. Through GeneCards and Ensembl databases, we improved the annotated information of autism-related genes, including mutated genes, mutation types and relevant supporting documents, and finally obtained 1,905 autism genes (Table S1). Imprinted genes information could acquire from Geneimprint (http://www.geneimprint.com/site/genes-by-species), in which all imprinted genes are listed. The normal human microarray data information is available in NCBI (registry number: GSE25219, https://www.ncbi.nlm.nih.gov/geo/query/acc.cgi).

## Abbreviations

AGP: Autism Genome Project
AGRE: Autism Genetic Resource Exchange
AS: Angelman Syndrome
ASD: Autism Spectrum Disorder
BWS: Beckwith-Wideman Syndrome
CAD: Computer Aided Design
CGH: Comparative Genomic Hybridization
CNS: Central Nervous System
CNV: Copy Number Variations
DMR: Differentially Methylated Region
FC: Fold Change
GD: Graphic Display
GEO: Gene Expression Omnibud
GO: Gene Ontology
GWAS: Genome-wide Association Study
HGMD: The Human Gene Mutation Database
ICR: Imprinting Control Region
IUGR: Intrauterine Growth Restriction
KEGG: Kyoto Encyclopedia of Genes and Genomes
MRI: Magnetic Resonance Imagining
NCBI: National Center for Biotechnology Information
ORA: Over-representation Analysis
PET: Positron Emission Tomography
PWS: Prader-Willi Syndrome
SFARI: The Simons Foundation Autism Research Initiative
SNP: Single Nucleotide Polymorphism
WES: Whole Exome Sequencing
WGCNA: Weighted Gene Coexpression Network Analysis
