# Supplementary figures and images for: Potential role of genomic imprinted genes and brain developmental related genes in autism
Source: BMC Med Genomics. 2020 Mar 26;13:54. doi: 10.1186/s12920-020-0693-2 (PMC7099798; doi:10.1186/s12920-020-0693-2)

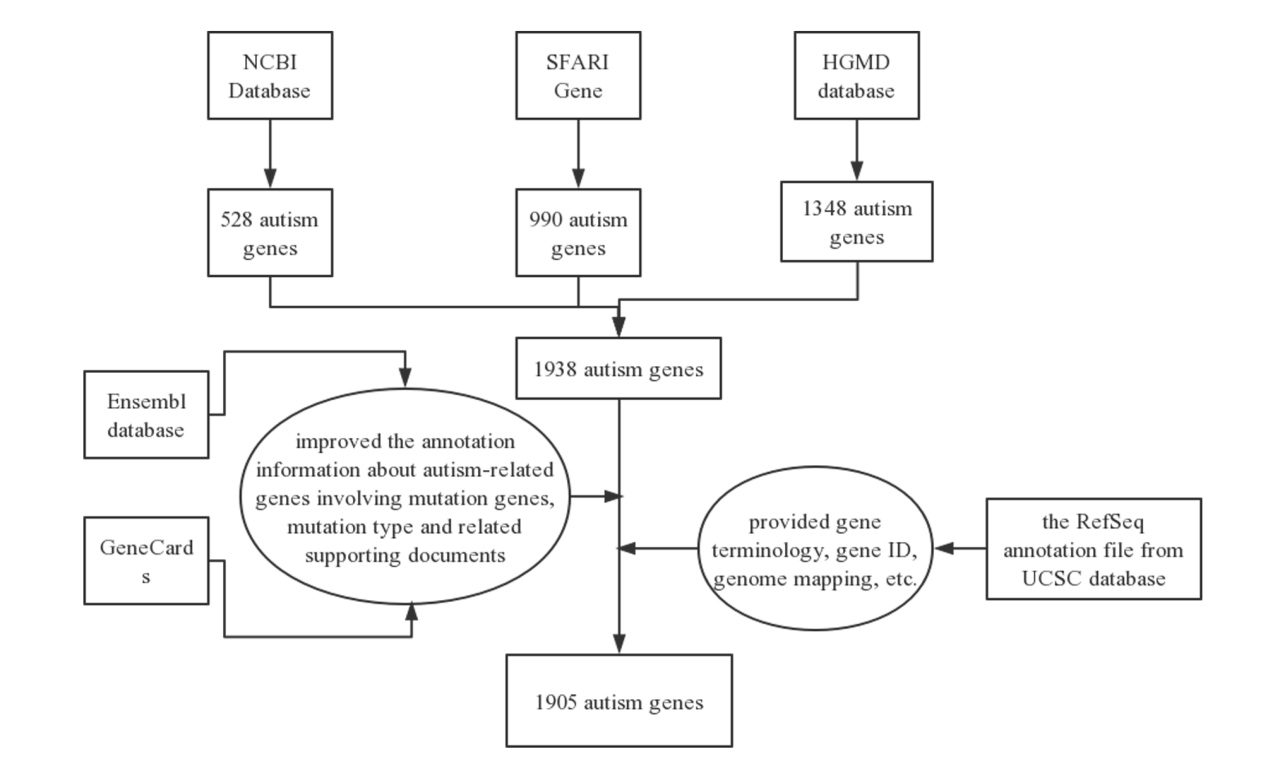

Supplement: Supplementary file 1 — Additional file 1: Figure S1. The screening process of autism-related genes. The 512 autism-related genes were collected from NCBI using retrieval condition: “autism” AND “Homo sapiens” [porgn:__txid9606]. We collected 990 and 1348 autism-related genes from SFARI Gene database and HGMD database, respectively. We annotated these genes with the information of gene terminology, gene ID, genome mapping, mutation genes, mutation type and related supporting documents, etc., from GeneCards and Ensembl databases. [file 12920_2020_693_MOESM1_ESM.docx]

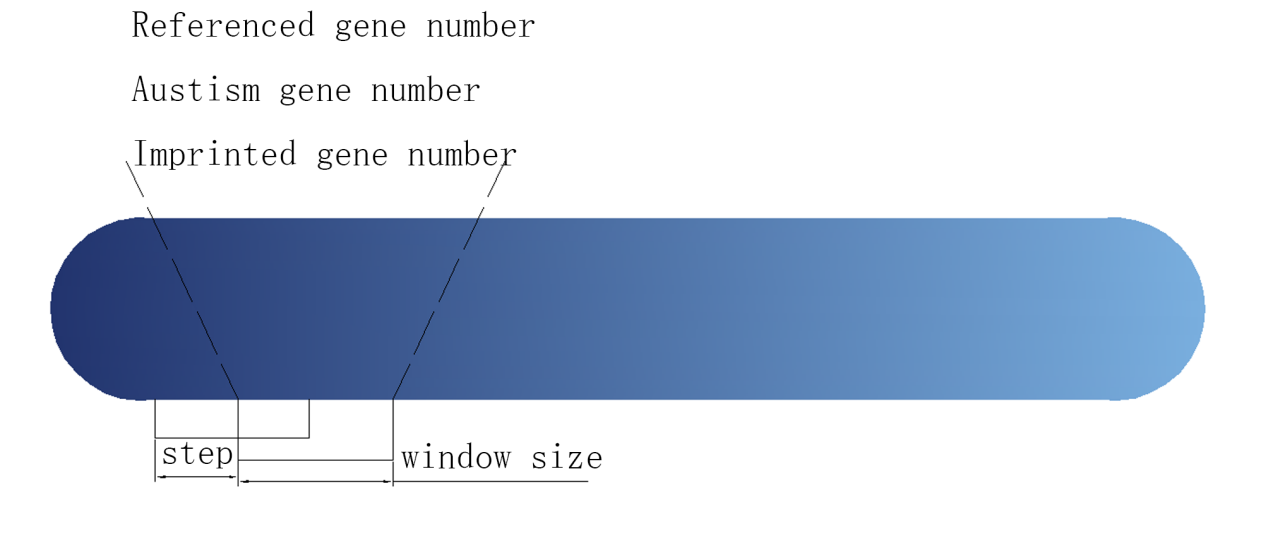

Supplement: Supplementary file 2 — Additional file 2: Figure S2. Sliding window schematic diagram. The length of each chromosome was known and the position information of the genes on the chromosomes has been annotated. Setting a w size window, which slided from the starting position to the end on the chromosomes, step by step, until whole chromosome was traversed. [file 12920_2020_693_MOESM2_ESM.docx]

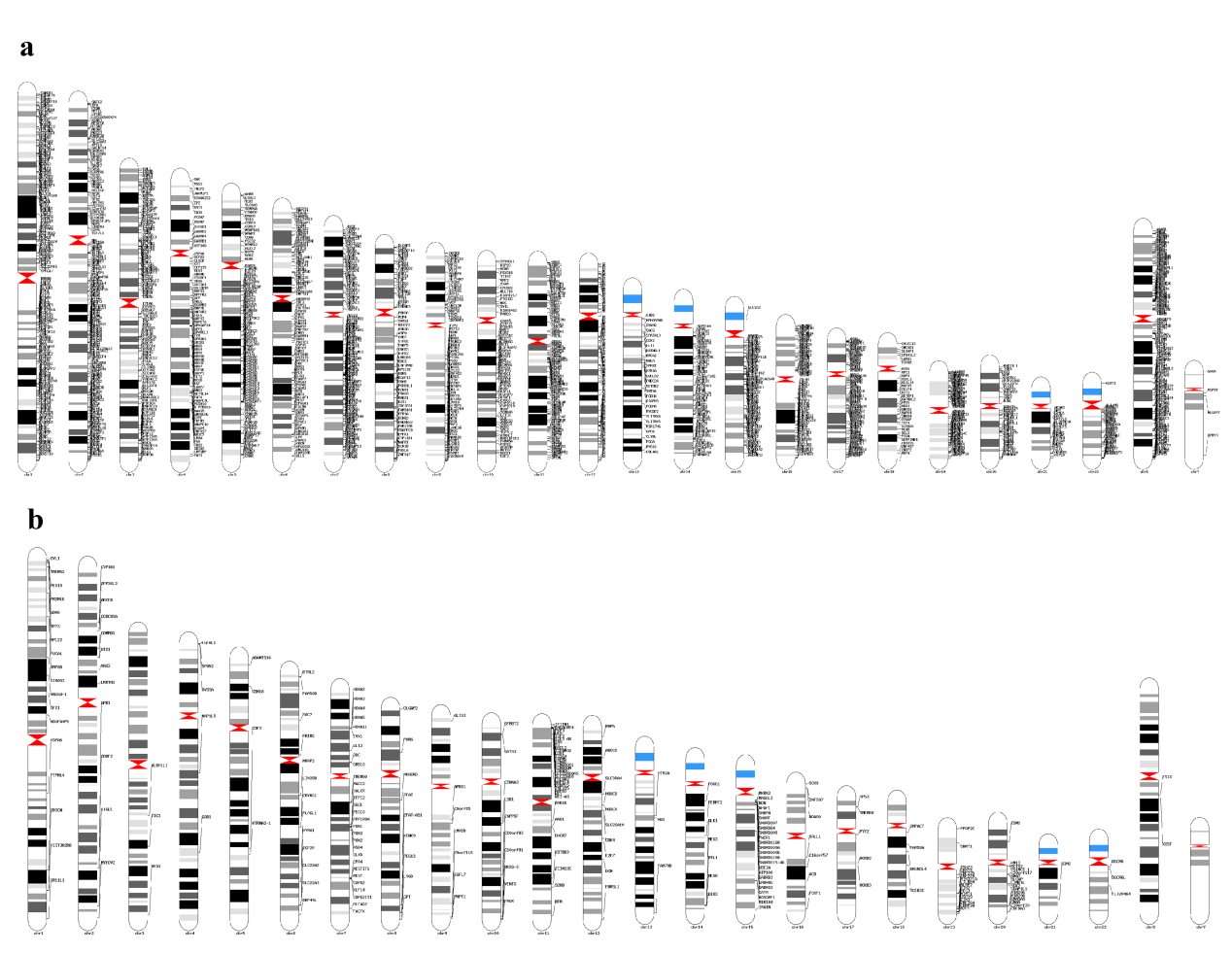

Supplement: Supplementary file 3 — Additional file 3: Figure S3. The distribution of imprinted genes and autism-related genes on chromosome 1~Y. a. Distribution of imprinted genes on chromosomes 1~ − 22 and chromosomes X - Y. b. Distribution of autism-related genes on chromosome 1~ − 22 and chromosomes X - Y. [file 12920_2020_693_MOESM3_ESM.docx]

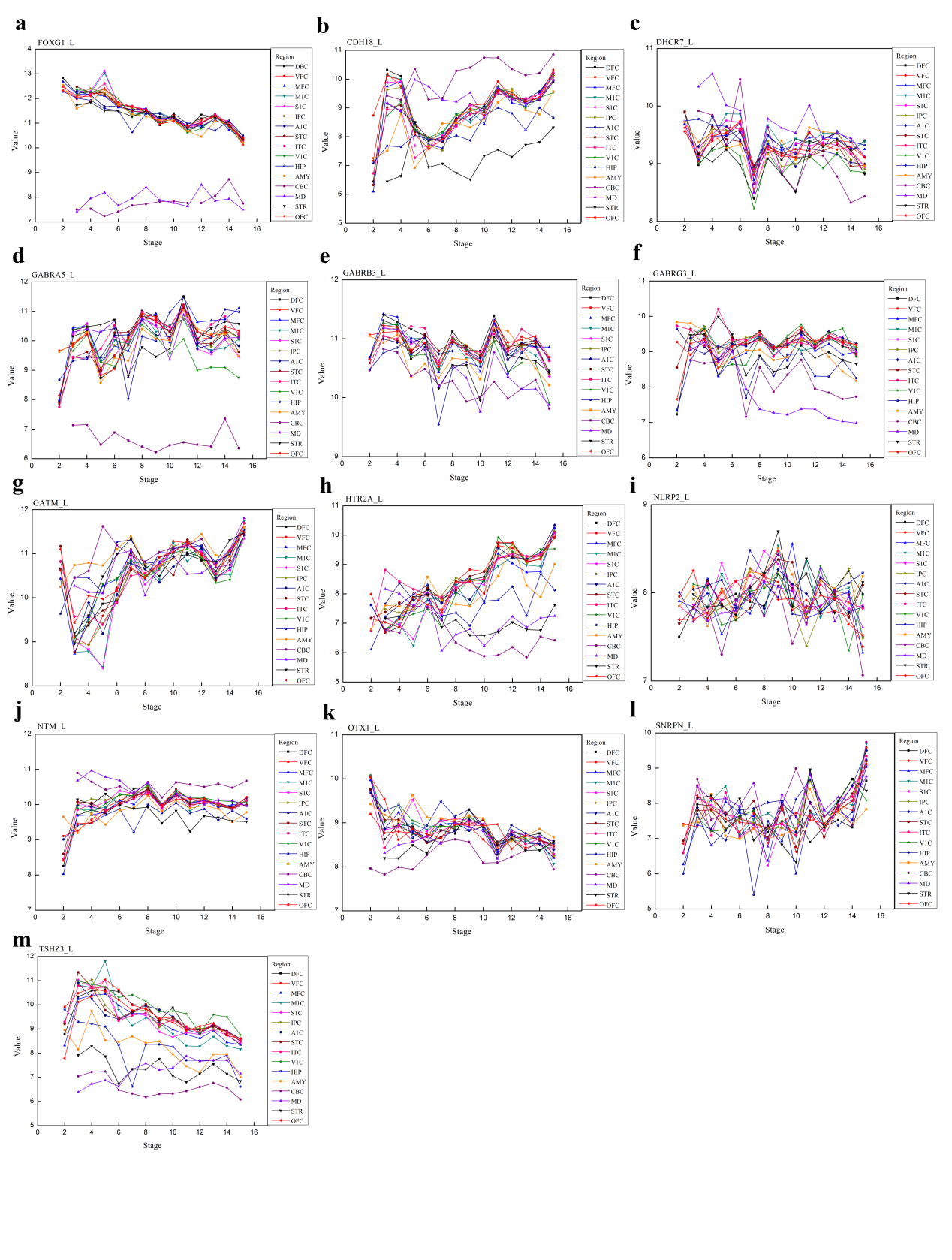

Supplement: Supplementary file 4 — Additional file 4: Figure S4. Expression trajectory plot of 13 common genes. a-c. The first line from left to right is CDH18, DHCR7 and FOXG1, respectively. d-f. The second line from left to right is GABRA5, GABRB3 and GABRG3, respectively. g-i. The third is GATM, HTR2A and NLRP2, respectively. j-l. The fourth line is NTM, OTX1 and SNRPN, respectively. m. The last line is TSHZ3. X axis: the 15 developmental periods; Y axis: the expression levels of different genes; The different colored lines indicate the expression of the same gene in different brain regions. [file 12920_2020_693_MOESM4_ESM.docx]
