# Supplementary material for: Potential role of genomic imprinted genes and brain developmental related genes in autism
Source: BMC Med Genomics. 2020 Mar 26;13:54. doi: 10.1186/s12920-020-0693-2 (PMC7099798; doi:10.1186/s12920-020-0693-2)
Supplement: Supplementary file 6 — Additional file 6: Table S2. The name of brain and cortical areas. [file 12920_2020_693_MOESM6_ESM.docx]

Table S2 The name of brain and cortical areas

|  | 1-2 Stage | 3-15 Stage | |
| --- | --- | --- | --- |
| Neocortex NCX（11） | Frontal cortex (FC) | Orbital frontal cortex | OFC |
|  |  | Dorsolateral prefrontal cortex | DFC |
|  |  | Ventral prefrontal cortex | VFC |
|  |  | Medial prefrontal cortex | MFC |
|  |  | Primary motor cortex | M1C |
|  | Parietal cortex (PC) | Primary sensory cortex | S1C |
|  |  | Inferior parietal cortex | IPC |
|  | Temporal cortex (TC) | Primary auditory cortex | A1C |
|  |  | Superior temporal cortex | STC |
|  |  | Inferior temporal cortex | ITC |
|  | Occipital cortex (OC) | Primary visual cortex | V1C |
| - | [Hippocampus](#/javascript:;) (HIP) | [Hippocampus](#/javascript:;) | HIP |
| - |  | Amygdala | AMY |
| - | The ventral forebrain (VF) | striatum | STR |
| - | Medial ganglionic eminence (MGE) |  |  |
| - | Lateral ganglionic eminence (LGE) |  |  |
| - | Caudal ganglionic eminence (CGE) |  |  |
| - | Diencephalon (DIE) | Mediodorsal thalamus | MD |
| - | Dorsal thalamus (DTH) | - | - |
|  | Cerebellar cortex (CBC) | Cerebellar cortex | CBC |
