# Supplementary material for: Potential role of genomic imprinted genes and brain developmental related genes in autism
Source: BMC Med Genomics. 2020 Mar 26;13:54. doi: 10.1186/s12920-020-0693-2 (PMC7099798; doi:10.1186/s12920-020-0693-2)
Supplement: Supplementary file 7 — Additional file 7: Table S3. The definition of human development stage. [file 12920_2020_693_MOESM7_ESM.docx]

Table S3 The definition of human development stage

| Stage | Definition | Age |
| --- | --- | --- |
| 1 | Embryonic period | 4PCW-8PCW |
| 2 | Early fetal period | 8PCW-10PCW |
| 3 | Early fetal period | 10PCW-13PCW |
| 4 | Early middle fetal | 13PCW-16PCW |
| 5 | Early middle fetal | 16PCW-19PCW |
| 6 | Fetal middle | 19PCW-24PCW |
| 7 | Fetal late | 24PCW-38PCW |
| 8 | Newborns and early infants | 0M-6M |
| 9 | [infancy](E:/Dict/8.5.1.0/resultui/html/index.html#/javascript:;) [stage](E:/Dict/8.5.1.0/resultui/html/index.html#/javascript:;) | 6M-12M |
| 10 | [preschool](E:/Dict/8.5.1.0/resultui/html/index.html#/javascript:;) [period](E:/Dict/8.5.1.0/resultui/html/index.html#/javascript:;) | 1Y-6Y |
| 11 | [childhood](E:/Dict/8.5.1.0/resultui/html/index.html#/javascript:;) | 6Y-12Y |
| 12 | [pubertas](E:/Dict/8.5.1.0/resultui/html/index.html#/javascript:;) | 12Y-20Y |
| 13 | adult stage | 20Y-40Y |
| 14 | middle age | 40Y-60Y |
| 15 | Old age | ＞60Y |

PCW: post-conceptional weeks; M: postnatal months; Y: postnatal years.
