# Supplementary material for: Potential role of genomic imprinted genes and brain developmental related genes in autism
Source: BMC Med Genomics. 2020 Mar 26;13:54. doi: 10.1186/s12920-020-0693-2 (PMC7099798; doi:10.1186/s12920-020-0693-2)
Supplement: Supplementary file 10 — Additional file 10: Table S6. The common genes of normal human brain differentially expressed genes, imprinted genes and autism-related genes. [file 12920_2020_693_MOESM10_ESM.docx]

| Gene Symbol | Entrez Id | Species | Gene Name | Mutation Type | Evidence | Protein Symbol | Protein | Function |
| --- | --- | --- | --- | --- | --- | --- | --- | --- |
| TSHZ3 | 57616 | Homo sapiens | teashirt zinc finger homeobox 3 (TSHZ3) | mice experiment | TSHZ3 deletion causes an autism syndrome and defects in cortical projection neurons | Q63HK5-TSH3_HUMAN | Teashirt homolog 3 | Transcriptional regulator involved in developmental processes. Function in association with APBB1, SET and HDAC factors as a transcriptional repressor, that inhibits the expression of CASP4. TSHZ3-mediated transcription repression involves the recruitment of histone deacetylases HDAC1 and HDAC2. Associates with chromatin in a region surrounding the CASP4 transcriptional start site(s) (PubMed: 19343227). Regulates the development of neurons involved in both respiratory rhythm and airflow control. Promotes maintenance of nucleus ambiguus (nA) motoneurons, which govern upper airway function, and establishes a respiratory rhythm generator (RRG) activity compatible with survival at birth. Involved in the differentiation of the proximal uretic smooth muscle cells during developmental processes. Involved in the up-regulation of myocardin, that directs the expression of smooth muscle cells in the proximal ureter (By similarity). Involved in the modulation of glutamatergic synaptic transmission and long-term synaptic potentiation (By similarity) |
| GABRG3 | 2567 | Homo sapiens | gamma-aminobutyric acid type A receptor gamma3 subunit (GABRG3) | GWAS | Nominal association between the GABRG3 gene and ASD has been observed in a Caucasian cohort (Menold et al., 2001) and, more recently, a Chinese ASD cohort (Wang et al., 2018); however, other studies have failed to show association between this gene and ASD (McCauley et al., 2004; Ma et al., 2005; Tochigi et al., 2007; Kelemenova et al., 2010; Mahdavi et al., 2018). Yang et al., 2017 found association between the GABRG3 SNP rs208129 and symptom-based phenotypes, as evaluated by CARS and ABC, in a cohort of 99 Chinese Han children and adolescents with ASD. Wang et al., identified a rare missense variant that was predicted to be deleterious (p.Val233Met) that was statistically enriched in Han Chinese ASD cases compared to controls (9/512 ASD cases vs. 2/575 controls; p = 0.020). | Q99928-GBRG3_HUMAN | Gamma-aminobutyric acid receptor subunit gamma-3 | GABA, the major inhibitory neurotransmitter in the vertebrate brain, mediates neuronal inhibition by binding to the GABA/benzodiazepine receptor and opening an integral chloride channel. |
| GABRB3 | 2562 | Homo sapiens | gamma-aminobutyric acid type A receptor beta3 subunit (GABRB3) | rare variants in ASD patients | Genetic analysis of GABRB3 as a candidate gene of autism spectrum disorders | P28472-GBRB3_HUMAN | Gamma-aminobutyric acid receptor subunit beta-3 | Ligand-gated chloride channel which is a component of the heteropentameric receptor for GABA, the major inhibitory neurotransmitter in the brain (PubMed: 18514161, PubMed: 22303015, PubMed: 26950270, PubMed: 22243422, PubMed: 24909990). Plays an important role in the formation of functional inhibitory GABAergic synapses in addition to mediating synaptic inhibition as a GABA-gated ion channel (PubMed: 25489750). The gamma2 subunit is necessary but not sufficient for a rapid formation of active synaptic contacts and the synaptogenic effect of this subunit is influenced by the type of alpha and beta subunits present in the receptor pentamer (By similarity). The alpha1/beta3/gamma2 receptor exhibits synaptogenic activity (PubMed: 25489750). The alpha2/beta3/gamma2 receptor shows very little or no synaptogenic activity (By similarity). Functions also as histamine receptor and mediates cellular responses to histamine (PubMed: 18281286). Plays an important role in somatosensation and in the production of antinociception (By similarity) |
| GATM | 2628 | Homo sapiens | glycine amidinotransferase (GATM) | ASD patients | An analysis of the clinical, biochemical, and molecular findings in 27 patients with GAMT deficiency determined that 21 of these patients (78%) were autistic, hyperactive, and self-injurious (Mercimek-Mahmutoglu et al., 2006). | P50440-GATM_HUMAN | Glycine amidinotransferase, mitochondrial | Catalyzes the biosynthesis of guanidinoacetate, the immediate precursor of creatine. Creatine plays a vital role in energy metabolism in muscle tissues. May play a role in embryonic and central nervous system development. May be involved in the response to heart failure by elevating local creatine synthesis. |
| SNRPN | 6638 | Homo sapiens | small nuclear ribonucleoprotein polypeptide N (SNRPN) | rare single gene mutation | This gene was identified as an ASD candidate gene following the identification of a balanced chromosomal abnormality (BCA) leading to gene disruption in an ASD case (Talkowski et al., 2012). | P63162-RSMN_HUMAN | Small nuclear ribonucleoprotein-associated protein N | May be involved in tissue-specific alternative RNA processing events. |
| OTX1 | 5013 | Homo sapiens | orthodenticle homeobox 1 (OTX1) | GWAS | In OTX1, rs2018650 and rs13000344 were associated with autism in ASD-CARC cohorts (P (FDR)=8.65 x 10 (-7) and 6.07 x 10 (5), respectively), AGRE cohort (P (FDR)=0.0034 and 0.015, respectively) and the combined families (P (FDR)=2.34 x 10 (-9) and 0.00017, respectively); | P32242-OTX1_HUMAN | Homeobox protein OTX1 | Probably plays a role in the development of the brain and the sense organs. Can bind to the BCD target sequence (BTS): 5'-TCTAATCCC-3'. |
| DHCR7 | 1717 | Homo sapiens | 7-dehydrocholesterol reductase (DHCR7) | mice experiment | Behavioral and serotonergic response changes in the Dhcr7-HET mouse model of Smith-Lemli-Opitz syndrome. | Q9UBM7-DHCR7_HUMAN | 7-dehydrocholesterol reductase | Production of cholesterol by reduction of C7-C8 double bond of 7-dehydrocholesterol (7-DHC) |
| CDH18 | 1016 | Homo sapiens | cadherin 18 (CDH18) | de novo translocation deleting CDH18 in ASD | Structural Variation of Chromosomes in Autism Spectrum Disorder | Q13634-CAD18_HUMAN | Cadherin-18 | Cadherins are calcium-dependent cell adhesion proteins. They preferentially interact with themselves in a homophilic manner in connecting cells; cadherins may thus contribute to the sorting of heterogeneous cell types. |
| FOXG1 | 2290 | Homo sapiens | forkhead box G1 (FOXG1) | WGS | FOXG1-dependent dysregulation of GABA/glutamate neuron differentiation in autism spectrum disorders | P55316-FOXG1_HUMAN | Forkhead box protein G1 | Transcription repression factor which plays an important role in the establishment of the regional subdivision of the developing brain and in the development of the telencephalon. |
| GABRA5 | 2558 | Homo sapiens | gamma-aminobutyric acid type A receptor alpha5 subunit (GABRA5) | Rare Single Gene Mutation, Genetic Association, Functional | Reduced GABRA5 mRNA and protein levels were observed in post-mortem brain tissue from ASD cases compared to controls (Fatemi et al., 2010). Deletion of GABRA5 in mice resulted in autism-related behaviors including reduced social interactions, reduced ultrasonic vocalizations, and increased self-grooming (Zurek et al., 2016) | P31644-GBRA5_HUMAN | Gamma-aminobutyric acid receptor subunit alpha-5 | GABA, the major inhibitory neurotransmitter in the vertebrate brain, mediates neuronal inhibition by binding to the GABA/benzodiazepine receptor and opening an integral chloride channel. |
| NLRP2 | 55655 | Homo sapiens | NLR family pyrin domain containing 2 (NLRP2) | WES of ASD patients | Whole exome sequencing reveals inherited and de novo variants in autism spectrum disorder: a trio study from Saudi families | Q9NX02-NALP2_HUMAN | NACHT, LRR and PYD domains-containing protein 2 | Suppresses TNF- and CD40-induced NFKB1 activity at the level of the IKK complex, by inhibiting NFKBIA degradation induced by TNF. When associated with PYCARD, activates CASP1, leading to the secretion of mature proinflammatory cytokine IL1B. May be a component of the inflammasome, a protein complex which also includes PYCARD, CARD8 and CASP1 and whose function would be the activation of proinflammatory caspases. |
| NTM | 50863 | Homo sapiens | Neurotrimin (NTM) | Sequencing of ASD patients | 11q24.2-25 micro-rearrangements in autism spectrum disorders: Relation to brain structures | Q9P121-NTRI_HUMAN | Neurotrimin | Neural cell adhesion molecule. |
| HTR2A | 3356 | Homo sapiens | 5-hydroxytryptamine receptor 2A (HTR2A) | GWAS | Possible genetic association has been found between the HTR2A gene and autism in a US population cohort, although none were significant in a TDT test (Veenstra-VanderWeele et al., 2002) | P28223-5HT2A_HUMAN | 5-hydroxytryptamine receptor 2A | G-protein coupled receptor for 5-hydroxytryptamine (serotonin) (PubMed: 1330647, PubMed: 18703043, PubMed: 19057895). Also functions as a receptor for various drugs and psychoactive substances, including mescaline, psilocybin, 1-(2, 5-dimethoxy-4-iodophenyl)-2-aminopropane (DOI) and lysergic acid diethylamide (LSD) (PubMed: 28129538). Ligand binding causes a conformation change that triggers signaling via guanine nucleotide-binding proteins (G proteins) and modulates the activity of down-stream effectors (PubMed: 28129538). Beta-arrestin family members inhibit signaling via G proteins and mediate activation of alternative signaling pathways (PubMed: 28129538). Signaling activates phospholipase C and a phosphatidylinositol-calcium second messenger system that modulates the activity of phosphatidylinositol 3-kinase and promotes the release of Ca^2+^ ions from intracellular stores (PubMed: 18703043, PubMed: 28129538). Affects neural activity, perception, cognition and mood (PubMed: 18297054). Plays a role in the regulation of behavior, including responses to anxiogenic situations and psychoactive substances. Plays a role in intestinal smooth muscle contraction, and may play a role in arterial vasoconstriction. |
